# Frailty Assessed Using the FRAIL-NH Scale and its Associations with Long-Term Care Needs in Residents of Japanese Nursing Homes: A Multicenter Cross-Sectional Study

**DOI:** 10.31662/jmaj.2025-0396

**Published:** 2025-11-28

**Authors:** Shota Hamada, Rumiko Tsuchiya-Ito, Shin J. Liau, Yukari Hattori, J. Simon Bell, Nobuo Sakata

**Affiliations:** 1 Department of Pharmacoepidemiology, School of Pharmacy, Tokyo University of Pharmacy and Life Sciences, Tokyo, Japan; 2 Research Department, Institute for Health Economics and Policy, Association for Health Economics Research and Social Insurance and Welfare, Tokyo, Japan; 3 Department of Health Services Research, Faculty of Medicine, University of Tsukuba, Tsukuba, Japan; 4 Research Department, Dia Foundation for Research on Ageing Societies, Tokyo, Japan; 5 Centre for Medicine Use and Safety, Faculty of Pharmacy and Pharmaceutical Sciences, Monash University, Melbourne, Australia; 6 National Health and Medical Research Council (NHMRC) Centre of Research Excellence in Frailty and Healthy Ageing, Adelaide, Australia; 7 Department of Geriatric Medicine, Graduate School of Medicine, The University of Tokyo, Tokyo, Japan; 8 Heisei Medical Welfare Group Research Institute, Tokyo, Japan

**Keywords:** FRAIL-NH, frailty, long-term care needs, nursing home

## Abstract

**Introduction:**

Frailty is often considered a pre-disability overall health state. However, even among older adults with disability, assessing frailty can be crucial for providing appropriate support and care services that maintain or promote their remaining independent abilities. The FRAIL-NH scale was developed to assess frailty in nursing home residents and has been shown to be useful in predicting their prognosis. We aimed to determine the prevalence and degree of frailty assessed by the FRAIL-NH scale among nursing home residents in Japan and investigate the associations between frailty status and long-term care (LTC) needs.

**Methods:**

A cross-sectional study was conducted in four nursing homes in the Tokyo metropolitan area of Japan, 2020. Frailty status was assessed using the 7-item FRAIL-NH scale Japanese version: non-frail (0-1 points), frail (2-5 points), and most-frail (6-14 points). Levels of LTC needs at the latest LTC needs certification were obtained from electronic health records. Age- and sex-adjusted multivariable logistic regression analyses were conducted to evaluate the associations between frailty status and the level of LTC needs.

**Results:**

Among 372 residents, 20.7% and 69.9% were frail and most-frail, respectively. Adjusted odds ratios (95% confidence intervals) for being either frail or most-frail were 3.62 (1.31-10.01) and 3.69 (1.14-11.94) for LTC needs levels 4 and 5, respectively, compared to level 3. Similarly, adjusted odds ratios (95% confidence intervals) for most-frail were 3.09 (1.77-5.38) and 6.38 (3.13-13.03) for LTC needs levels 4 and 5, respectively.

**Conclusions:**

Most nursing home residents were assessed as being frail or most-frail. Frailty was strongly associated with LTC needs, indicating important resource implications for care services. Moreover, assessing frailty with the FRAIL-NH scale may support medical and care-related decision-making.

## Introduction

Japan has among the world’s highest proportion of older people. This means that frailty presents an important public health challenge ^(1), (2), (3)^. Frailty is characterized by increased susceptibility to stressors, resulting from a cumulative decline in physiological systems ^(4)^. Stressors can cause disproportionate changes in the health status of frail older adults, and may lead to adverse consequences, such as falls, functional impairment, hospitalizations, and mortality ^(4), (5), (6)^. Frailty is also characterized by its potential reversibility and possible attenuation through appropriate interventions ^(5), (7)^.

Frailty is often considered a pre-disability overall health state. However, even among older adults with disability, assessing frailty can be crucial for providing appropriate support and care services that maintain or promote their remaining independent abilities. The number of nursing home residents in Japan has more than doubled over the past 15 years. Knowledge about the prevalence of frailty and the association with long-term care (LTC) needs is important to inform health policy and planning in the context of changing population demographics and models of aged care.

Frailty assessment can guide treatment optimization and promote person-centered care among frail older adults ^(6), (8), (9)^. This may assist with delaying or potentially reversing decline in capacity ^(10)^. A recent cohort study found that, while 32.3% of nursing home residents had no change to their frailty status after 12 months of follow-up, 13.7% had an improvement in their frailty status during the same period ^(11)^. Frailty screening in nursing homes may help identify residents who would benefit from a review of treatment benefits and risks in light of changing goals of care ^(12)^. A systematic review reported that the pooled prevalence estimate of frailty among 1,373 nursing home residents from nine studies was 52.3% with a wide variation from 19.0% to 75.6% ^(13)^. A more recent systematic review also demonstrated wide variability in the prevalence of frailty measured using the FRAIL-NH scale: 15.1%-79.5% for frail and 28.5%-75.0% for most-frail ^(14)^.

The FRAIL-NH scale ^(15)^ is a practical frailty screening tool specific for nursing homes where resources such as time, equipment, and clinical expertise may be limited. The FRAIL-NH scale consists of seven potentially reversible domains, including (1) fatigue, (2) resistance, (3) ambulation, (4) incontinence or illness, (5) loss of weight, (6) nutritional approach, and (7) help with dressing. Previous studies have shown that the FRAIL-NH scale (phenotype model) had good agreements with other scales, such as the Frailty Index (cumulative deficit model) ^(16), (17)^. Frailty measured using the FRAIL-NH scale has been associated with various adverse outcomes including mortality ^(16), (18), (19), (20)^.

We have previously reported the prevalence of frailty among 333 nursing home residents with valid medication data, as part of cross-national studies on medication use, stratified by frailty level ^(21), (22), (23)^. We used the recently developed Japanese version of the FRAIL-NH scale ^(24)^ to assess frailty in these studies. However, the distribution of FRAIL-NH scores and the overall prevalence and degree of frailty have not yet been published in a more comprehensive population, regardless of medication data validity. In addition, it is important to know how frailty is related to level of LTC needs certified under the public LTC insurance system in Japan ^(25), (26)^. The objective of this study was to (1) determine the prevalence and degree of frailty among nursing home residents in Japan, and (2) investigate the associations between frailty status and LTC needs.

## Materials and Methods

### Study setting and participants

We conducted cross-sectional analyses of a prospective cohort study involving residents of four nursing homes (called Special Nursing Homes or *Tokuyo*) located in Tokyo or Kanagawa prefecture (adjacent to Tokyo). In general, Japanese nursing homes accommodate approximately 25% of older adults with high LTC needs (i.e., LTC needs levels 3 or higher) ^(27), (28)^, and have the largest capacity among the different types of LTC or residential facilities available for older adults.

Residents in the participating nursing homes were invited to participate in the study by nursing home staff. The total bed capacity of the four facilities was 514, and all residents were considered eligible because no exclusion criteria were applied. Prior to data collection, each facility determined a feasible target number of participants, and the sum of these targets was set at 440 residents. Recruitment was conducted until the target number for each facility was reached or until the end of the data collection period. Residents were included if the individual or their representative provided written informed consent. All data were collected after anonymization at each nursing home. Baseline data (age, sex, height, body weight, medical history, and disability levels which were assessed at the latest LTC needs certification ^(29)^) were collected from electronic health records. The study protocol was approved by the ethical review board of the Institute for Health Economics and Policy (R2-002). The study was performed in accordance with the Declaration of Helsinki and the current ethical guidelines.

### Frailty assessment

Residents’ frailty status was assessed using the FRAIL-NH scale Japanese version by nurses, physiotherapists, occupational therapists, and other nursing home staff between October and December 2020 (Table S1; Additional file; back-translated version) ^(24)^. Clear written instructions were provided to guide frailty assessment based on feedback obtained during development of the FRAIL-NH scale Japanese version ^(24)^. For the fourth FRAIL-NH domain, we selected ‘incontinence’ rather than ‘illness’. This was because the number of prescribed medications may not be a good proxy for illness due to medication deprescribing in residents with advanced dementia and at the end-of-life ^(30)^. A score of 0, 1, or 2 was assigned to each FRAIL-NH item, with a maximum total score of 14 points. Given that the optimal FRAIL-NH cut-off point has not been established for Japanese nursing home residents, we adopted the frequently used cut-off for frailty categorization as follows: 0-1, non-frail; 2-5, frail; and ≥6, most-frail, based on a recent systematic review on the FRAIL-NH scale ^(14)^. A sensitivity analysis was also conducted on a two-category frailty using another frequently used cut-off point of ≥8 ^(14)^.

### LTC needs assessment

Data were extracted on the level of LTC needs documented in each resident’s latest LTC needs certification. The LTC need certification is undertaken using a 74-item survey on physical and mental conditions and additional examinations, including the use of specific medical procedures. Through the certification process, levels of LTC are determined based on the estimated time needed for LTC, as well as physical or cognitive independence levels. Generally, individuals are entitled to receive LTC services after their LTC needs are certified through a nationally standardized LTC needs certification scheme under the public LTC insurance system in Japan ^(25), (26)^. Individuals are eligible to renew the certification every 2 years or more frequently if necessary. The level of LTC needs is ranked from 1 to 5, where 5 represents the highest need for LTC services.

### Statistical analyses

Descriptive statistics were used to summarize the demographic and clinical characteristics of the study participants. The prevalence of frailty (non-frail, frail, and most-frail) was calculated for all residents, and stratified according to level of LTC needs. Multivariable logistic regression analyses were conducted to evaluate the associations between frailty status (outcome 1: both frail and most-frail; and outcome 2: most-frail only) and level of LTC needs, adjusted for age (<80, 80-89, and ≥90 years) and sex. For sensitivity analysis, we repeated the analysis with a two-category frailty based on another commonly used cut-off point that was different from the main analysis. All analyses were performed using Stata version 17 (Stata Corp., College Station, TX, USA).

## Results

### Characteristics of study participants

The study included 372 residents (85% of 440 target recruitment achieved). This shortfall mainly occurred because some residents had difficulty communicating and consent could not be obtained from their family representatives, or staff resources for conducting the survey were insufficient. The mean (±standard deviation) age of the participants was 86 (±7) years, and 72.6% were women (Table 1). Half of the participants had a length of stay of >1 year from the first admission to the nursing home. Among the participants, 65.5% had a normal body mass index, whereas 24.8% were underweight. The most common medical diagnosis was dementia, followed by fracture and stroke. The proportions of LTC needs levels 3, 4, and 5 were 25.3%, 41.1%, and 27.7%, respectively. Based on the disability levels reported in the latest certification of LTC needs, 73.4% were bedridden (“Independence degree of daily living for older adults with disability” rank B or C) and 67.7% had severe dementia (“Independence degree of daily living for older adults with dementia” rank III, IV, or M).

**Table 1.** Characteristics of Study Participants.

		n (%)
Age (years)	Mean ± SD	86.2 ± 7.1
	<80	54 (14.5)
	80-89	184 (49.5)
	≥90	134 (36.0)
Sex	Men	102 (27.4)
	Women	270 (72.6)
Days after the first admission to a facility	≤90	31 (8.3)
	91-180	52 (14.0)
	181-365	95 (25.5)
	>365	194 (52.2)
Body mass index (kg/m^2^)*	Mean ± SD	20.8 ± 3.6
	Underweight (<18.5)	92 (24.8)
	Normal range (18.5-24.9)	243 (65.5)
	Pre-obese to obese (≥25.0)	36 (9.7)
Comorbidities/History of diseases	Dementia	259 (69.6)
	Fractures	181 (48.7)
	Aspiration pneumonia	68 (18.3)
	Stroke	131 (35.2)
	Ischemic heart disease	45 (12.1)
	Atrial fibrillation	36 (9.7)
	Diabetes	93 (25.0)
	Cancer (active)	8 (2.2)
	Cancer (history)	57 (15.3)
Long-term care needs level	1 or 2	22 (5.9)
	3	94 (25.3)
	4	153 (41.1)
	5	103 (27.7)
Physical disability level (rank)^†^	J / A	99 (26.6)
	B1 / B2	203 (54.6)
	C1 / C2	70 (18.8)
Cognitive disability level (rank)^‡^	Independent / I	27 (7.3)
	IIa / IIb	93 (25.0)
	IIIa / IIIb	125 (33.6)
	IV / M	127 (34.1)

SD: standard deviation. *One missing data. ^†^Based on the “Independence degree of daily living for older adults with disability” are categorized as follows: Rank J: some disabilities, but daily living is mostly independent, capable of going outdoors unassisted; Rank A: indoor living is predominantly independent, but unable to go out without assistance; Rank B: some assistance needed for indoor living, also lies in bed for much of the daytime, although sitting is possible; and Rank C: bedridden all day, requires assistance with excretion/urination, meals, and dressing/undressing ^(29)^. ^‡^Based on the “Independence degree of daily living for older adults with dementia” are categorized as follows: Independent; Rank I: has some type of dementia, but almost independent in terms of daily living at home and in society; Rank II: some daily life-disturbing symptoms, behaviors and problems in communication seen but can lead daily life independently if watched over by someone; Rank III: daily life-disturbing symptoms, behaviors, and problems in communication that require assistance; Rank IV: daily life-disturbing symptoms, behaviors, and problems in communication that frequently require assistance; and Rank M: marked psychiatric symptoms/related symptoms or serious physical disorders that require expert management ^(29)^.

### Frailty assessment

The scores for each FRAIL-NH item are shown in Table 2. During frailty assessment, assessors expressed difficulties in judging the ‘fatigue’ domain for 70 residents due to severe dementia (n = 53), low level of consciousness (n = 14), and other reasons (n = 17; 14 of these were also due to severe dementia). These 70 residents were all assigned a score of 2 for the ‘fatigue’ domain to avoid potential underestimation of their frailty status. These results were subsequently categorized as most-frail based on their total FRAIL-NH scores. However, if a score of 0 rather than 2 was assigned instead, only three of the 70 residents would have been categorized as frail rather than most-frail.

**Table 2.** Scores of Individual Items in the FRAIL-NH Scale.

	0	1	2
Item name	n (%)	n (%)	n (%)
Fatigue*	212 (57.0)	45 (12.1)	115 (30.9)
Resistance	106 (28.5)	95 (25.5)	171 (46.0)
Ambulation	84 (22.6)	21 (5.6)	267 (71.8)
Incontinence^†^	70 (18.8)	63 (16.9)	239 (64.2)
Loss of weight	330 (88.7)	28 (7.5)	14 (3.8)
Nutritional approach	126 (33.9)	213 (57.3)	33 (8.9)
Help with dressing	47 (12.6)	53 (14.2)	272 (73.1)

^*^Two points were assigned to 70 participants (18.8%) whose “fatigue” scores were difficult to assess. ^†^“Incontinence” was assessed instead of “Illness.”

The scores of each domain of the FRAIL-NH scale are shown according to LTC needs levels (Table S2). Except for weight loss, which showed a similar distribution from LTC needs levels 3 or higher, scores in the other domains tended to shift toward higher scores as LTC needs levels increased. Moreover, even at the same LTC needs level, there was considerable variation in independent capacities.

**Figure 1.**
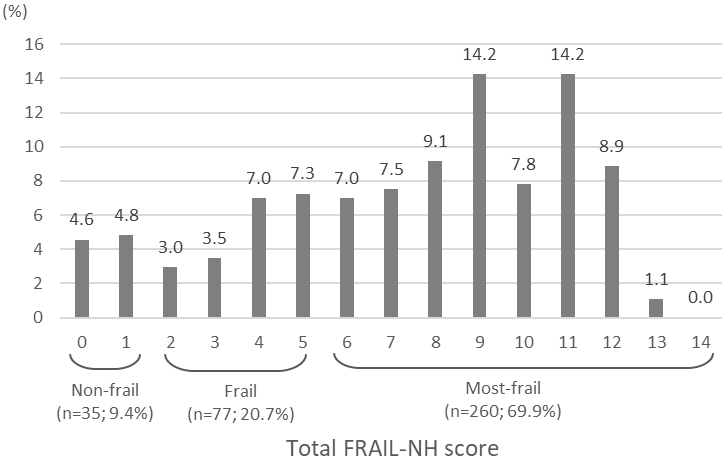
Distributions of total FRAIL-NH scores.

The median FRAIL-NH score was 8, and the interquartile range was 5-10. The distribution of the total scores is shown in Figure 1. Overall, the prevalence of non-frail, frail, and most-frail was 9.4% (n = 35), 20.7% (n = 77), and 69.9% (n = 260), respectively. If a score of 0 rather than 2 was assigned for fatigue in residents whose fatigue could not be assessed, the prevalence of frail and most-frail changed to 21.5% (n = 80) and 69.1% (n = 257), respectively. If the residents whose fatigue could not be well assessed (n = 70) were excluded from the analysis, the prevalence of non-frail, frail, and most-frail was 11.6% (n = 35), 24.5% (n = 74), and 63.9% (n = 193), respectively. In contrast, when the two-category frailty cut-off point of ≥8 was applied, 55.4% of residents were categorized as frail.

The characteristics of residents are summarized by frailty status in Table S3. Regarding comorbidities and disease history, dementia, aspiration pneumonia, and stroke tended to be more prevalent among residents with higher frailty.

### Associations between frailty status and level of LTC needs

**Figure 2.**
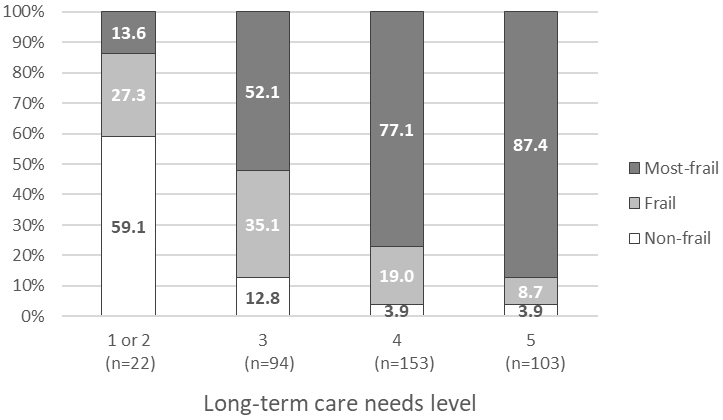
Prevalence of frailty according to levels of long-term care needs.

The higher the level of LTC needs, the higher the prevalence and degree of frailty (Figure 2). More than 95% of the residents with LTC needs levels 4 or 5 were either frail or most-frail. The associations between frailty status and level of LTC needs are shown in Table 3. Adjusted odds ratios (95% confidence intervals) for either frail or most-frail were 3.62 (1.31-10.01) and 3.69 (1.14-11.94) for LTC needs levels 4 and 5, respectively, compared to level 3. Similarly, adjusted odds ratios (95% confidence intervals) for most-frail were 3.09 (1.77-5.38) and 6.38 (3.13-13.03) for LTC needs levels 4 and 5, respectively. Results from the sensitivity analysis were similar to the main analysis (Table S4). Frailty status was not associated with age and sex.

**Table 3.** Associations between Frailty Status and Level of Long-term Care Needs.

		Outcomes			
		Frail/most-frail		Most-frail	
		Adjusted OR*	p Value	Adjusted OR*	p Value
Level of LTC needs	1 / 2	0.10 (0.04-0.29)	<0.001	0.14 (0.04-0.51)	0.003
	3	Ref	-	Ref	-
	4	3.62 (1.31-10.01)	0.013	3.09 (1.77-5.38)	<0.001
	5	3.69 (1.14-11.94)	0.029	6.38 (3.13-13.03)	<0.001

LTC: long-term care; OR: odds ratio; Ref: reference. *The models were adjusted for age group (<80, 80-89, and ≥90 years) and sex.

## Discussion

This was the first study to report the prevalence of frailty assessed using the FRAIL-NH scale in Japanese nursing home residents. The FRAIL-NH score was varied and peaked on the scores categorized as most-frail. Of the participants, 20.7% were categorized as frail and 69.9% as most-frail, resulting in 90.6% being either frail or most-frail. In addition, the prevalence and degree of frailty were associated with the level of LTC needs, independent of age and sex. Despite the high prevalence of frailty in nursing home residents, we believe that care addressing potentially reversible conditions should be provided within the maximum amount of services determined based on the level of LTC needs. Moreover, it would be important, especially for most-frail residents, to take frailty status into consideration to set residents’ goals of care and to make decisions of person-centered treatment approaches ^(6), (8), (9)^ because our previous research has suggested that Japanese clinicians may be less likely to modify medication regimens based on resident frailty status than clinicians in other countries ^(22), (23)^.

The prevalence of frailty in our study was higher than in most previous studies among nursing home residents conducted in other countries ^(13), (14)^. These differences may be attributed to the variations in characteristics of nursing home residents between different countries due to the availability of alternative care settings, such as home healthcare. In addition, our study cohort included most of the residents in the participating nursing homes. Several previous studies excluded residents with a short life expectancy ^(16), (31)^ or those who were deemed medically unstable ^(17), (31)^. This may have resulted in under-representation of frail residents in those studies.

This study demonstrated the high prevalence of frailty in Japanese nursing home residents, particularly among those with LTC needs levels 4 or 5. Considering that all the FRAIL-NH items, except for ‘fatigue’ and ‘loss of weight’, are included in the questionnaire used for the initial assessment of LTC needs ^(29)^, the associations between frailty and level of LTC needs were considered reasonable. In contrast, because LTC needs are comprehensively evaluated, including physical and cognitive functioning, some discrepancies between the measures shall exist. Although a recent Chinese cross-sectional study showed a linear relationship between frailty and LTC needs in community-dwelling older adults ^(32)^, our study adds new findings to the association between frailty and LTC needs in the nursing home setting.

This study has some limitations. First, we did not establish the inter-rater reliability of frailty assessment using the FRAIL-NH scale conducted by different assessors. However, all items except for the ‘fatigue’ domain were determined through objective observations. In this study, we assigned a score of 2 for the ‘fatigue’ domain for residents who were unable to report their fatigue status (e.g., residents with severe dementia, low consciousness). In future, use of a validated and objective assessment of fatigue by multiple observers may improve assessment of residents’ frailty status. Furthermore, the FRAIL-NH scale was designed to be used without the need for specialist training, and has been successfully used by a range of healthcare workers ^(14)^. Second, although we partly confirmed the robustness of our findings by applying commonly used FRAIL-NH cut-off points in this study, further studies are needed to determine the optimal FRAIL-NH cut-off point to identify frailty among nursing home residents in Japan. Third, the categorization of residents with LTC needs levels 4 or 5 as non-frail may appear counterintuitive. This may be due to the lag time between the latest certification for LTC needs and the timing of frailty assessment. It is important to perform timely assessments and regular reviews of residents’ health status, including frailty, upon admission to the nursing home, as well as during the stay, to determine the most appropriate treatment regimen that aligns with their current goals of care ^(12)^. Fourth, our study sample may not necessarily be representative or generalizable to all nursing home residents across Japan. The participating facilities were located only in urban areas. Regional differences in population characteristics and the availability of nursing home and home healthcare may lead to differences in resident characteristics. Therefore, to account for possible facility-level differences, research should also be conducted in other Japanese nursing homes.

As a direction for future research, further evidence is needed to establish the clinical utility of frailty assessment using the FRAIL-NH scale. Previous studies conducted overseas have reported associations between frailty assessed using this scale and outcomes such as hospitalization and mortality ^(14)^. Although such analyses were beyond the scope of the present study, it is important that future longitudinal research in Japanese nursing home residents examine whether frailty assessed using the FRAIL-NH scale predicts outcomes such as pneumonia, falls, deterioration in LTC needs levels, hospitalization, and mortality.

In conclusion, over 90% of Japanese nursing home residents were assessed as being frail or most-frail, measured using the FRAIL-NH scale. Frailty was strongly associated with LTC needs independent of age and sex, indicating important resource implications for the provision of aged care services in Japan and other countries with rapidly aging populations. Moreover, among nursing home residents, even with the same LTC needs level, there was considerable variation in independent capabilities. Therefore, assessing the important domains included in FRAIL-NH scale individually, or using FRAIL-NH scale comprehensively to capture frailty, may support medical and care-related decision-making.

## Article Information

### Author Contributions

Shota Hamada, J Simon Bell, and Nobuo Sakata conceptualized the study. Shota Hamada and Nobuo Sakata designed the study. Nobuo Sakata collected data. Shota Hamada performed the analyses. All authors interpreted the data. Shota Hamada drafted the manuscript. Rumiko Tsuchiya-Ito, Shin J Liau, Yukari Hattori, J Simon Bell, and Nobuo Sakata reviewed and revised the manuscript critically for important intellectual content. All authors have approved the final manuscript as submitted.

### Conflicts of Interest

Shota Hamada and Yukari Hattori belonged to an endowed chair funded by donations from Hakue Technology, PROUMED, Japan Bio Products, Towa Pharmaceutical, Yellow Eight, and Sugi Holdings at the time of this study. Shin J Liau was supported by a postgraduate research scholarship funded by Monash University, and the Australian Government Research Training Program Scholarship. Shin J Liau and J Simon Bell were supported by the National Health and Medical Research Council (NHMRC) Centre of Research Excellence in Frailty and Healthy Ageing. J Simon Bell was supported by the NHMRC Boosting Dementia Research Leadership Fellowship. J Simon Bell has received grant or consulting funds from the NHMRC, Medical Research Future Fund, Victorian Government Department of Health and Human Services, Dementia Australia Research Foundation, Yulgilbar Foundation, Aged Care Quality and Safety Commission, Australian Commission on Safety and Quality in Health Care, Dementia Centre for Research Collaboration, Pharmaceutical Society of Australia, Society of Hospital Pharmacists of Australia, GlaxoSmithKline Supported Studies Programme, Amgen, and several aged care provider organizations. All these funds were paid to Monash University. The other authors have no conflicts of interest to declare.

### Ethical Consideration

The study protocol was approved by the ethical review board of the Institute for Health Economics and Policy (R2-002). The study was performed in accordance with the Declaration of Helsinki and the current ethical guidelines.

### Data Availability Statement

The data that support the findings of this study are available from the corresponding author upon reasonable request.

### Informed Consent

Written informed consent was obtained from the study participant or their representative.

## Supplement

Supplementary Material
