# Supplementary material for: Frailty Assessed Using the FRAIL-NH Scale and its Associations with Long-Term Care Needs in Residents of Japanese Nursing Homes: A Multicenter Cross-Sectional Study
Source: JMA J. 2025 Nov 28;9(1):246–53. doi: 10.31662/jmaj.2025-0396 (PMC12889871; doi:10.31662/jmaj.2025-0396)
Supplement: Supplementary Material [file 2433-3298-9-1-0246-s001.pdf]

Table S1. Japanese Version of the FRAIL-NH Scale

No.	Item name (English original version)	0 point	1 point	2 points
1	Malaise ('Fatigue')	Absence of body fatigue	Presence of only body fatigue	Presence of body fatigue as well as loss of interest and motivation
2	Getting up/ Transferring ('Resistance')	Ability to transfer independently	Requires partial assistance to shift from the bed to a chair	Requires complete assistance to shift from the bed to a chair
3	Mobility ('Ambulation')	Independent	Requires a walker	Incapable/requires wheelchair
4-1	Incontinence[a]	None	Urinary incontinence	Fecal incontinence
4-2	Illness[a,b]	<5 types	5–9 types	≥10 types
5	Loss of weight	None	At least 5% weight loss in 3 months	At least 10% weight loss in 6 months
6	Nutrition/Feeding ('Nutritional approach')	Ability to eat normal foods independently	Requires processing of foods, such as mincing, or requires assistance in ingestion	Tube feeding
7	Dressing ('Help with dressing')	Independent	Requires clothes to be prepared	Requires assistance

a. Include either "Incontinence" or "Illness" to calculate the total score for the seven items.

b. Measured by number of medications

Permission was obtained for reprinting the back-translation of FRAIL-NH Japanese version from the Japan Geriatrics Society (No. R1658).

Sakata N, Hamada S, Tsuchiya R, Satake S. Development of FRAIL-NH scale Japanese version. Nippon Ronen Igakkai Zasshi. 2021;58:164-6. (in Japanese)

Table S2. Scores of individual items in the FRAIL-NH scale according to long-term care needs levels

	LTC needs level 1 or 2 (N = 22)			LTC needs level 3 (N = 94)		
	0	1	2	0	1	2
Item name	n (%)	n (%)	n (%)	n (%)	n (%)	n (%)
Fatigue[a]	19 (86.4)	1 (4.5)	2 (9.1)	62 (66.0)	15 (16.0)	17 (18.1)
Resistance	16 (72.7)	4 (18.2)	2 (9.1)	46 (48.9)	25 (26.6)	23 (24.5)
Ambulation	12 (54.5)	3 (13.6)	7 (31.8)	33 (35.1)	9 (9.6)	52 (55.3)
Incontinence[b]	14 (63.6)	5 (22.7)	3 (13.6)	28 (29.8)	26 (27.7)	40 (42.6)
Loss of weight	22 (100.0)	0 (0.0)	0 (0.0)	82 (87.2)	9 (9.6)	3 (3.2)
Nutritional approach	18 (81.8)	3 (13.6)	1 (4.5)	48 (51.1)	45 (47.9)	1 (1.1)
Help with dressing	13 (59.1)	5 (22.7)	4 (18.2)	13 (13.8)	26 (27.7)	55 (58.5)

	LTC needs level 4 (N = 153)			LTC needs level 5 (N = 103)		
	0	1	2	0	1	2
Item name	n (%)	n (%)	n (%)	n (%)	n (%)	n (%)
Fatigue[a]	90 (60.4)	24 (16.1)	35 (23.5)	42 (47.7)	7 (8.0)	39 (44.3)
Resistance	30 (19.6)	48 (31.4)	75 (49.0)	14 (13.6)	18 (17.5)	71 (68.9)
Ambulation	27 (17.6)	6 (3.9)	120 (78.4)	12 (11.7)	3 (2.9)	88 (85.4)
Incontinence[b]	22 (14.4)	23 (15.0)	108 (70.6)	6 (5.8)	9 (8.7)	88 (85.4)
Loss of weight	136 (88.9)	9 (5.9)	8 (5.2)	90 (87.4)	10 (9.7)	3 (2.9)
Nutritional approach	46 (30.1)	93 (60.8)	14 (9.2)	14 (13.6)	72 (69.9)	17 (16.5)
Help with dressing	15 (9.8)	20 (13.1)	118 (77.1)	6 (5.8)	2 (1.9)	95 (92.2)

LTC, long-term care

a. Two points were assigned to 70 participants (18.8%) whose ‘fatigue’ scores were difficult to assess: 1 (4.5%) for LTC needs level 1 or 2, 6 (6.4%) for LTC needs level 3, 22 (14.4%) for LTC needs level 4, and 41 (39.8%) for LTC needs level 5

b. ‘Incontinence’ was assessed instead of ‘Illness’.

Table S3. Comparisons of characteristics according to frailty status

		Frailty status		
		Non-frail (N = 35)	Frail (N = 77)	Most-frail (N = 260)
		n (%)	n (%)	n (%)
Age (years)	Mean ± SD	86.7 ± 7.2	85.2 ± 8.7	86.5 ± 6.6
	<80	4 (11.4)	16 (20.8)	34 (13.1)
	80–89	18 (51.4)	33 (42.9)	133 (51.2)
	≥90	13 (37.1)	28 (36.4)	93 (35.8)
Sex	Men	9 (25.7)	20 (26.0)	73 (28.1)
	Women	26 (74.3)	57 (74.0)	187 (71.9)
Days after the first admission to a facility	≤90	5 (14.3)	10 (13.0)	16 (6.2)
	91–180	7 (20.0)	15 (19.5)	30 (11.5)
	181–365	7 (20.0)	23 (29.9)	65 (25.0)
	>365	16 (45.7)	29 (37.7)	149 (57.3)
Body mass index (kg/m^2)	Mean ± SD	21.6 ± 4.1	21.4 ± 3.6	20.5 ± 3.4*
	Underweight (<18.5)	4 (11.4)	17 (22.1)	71 (27.4)
	Normal range (18.5–24.9)	26 (74.3)	50 (64.9)	167 (64.5)
	Pre-obese to obese (≥25.0)	5 (14.3)	10 (13.0)	21 (8.1)
Comorbidities/History of diseases	Dementia	19 (54.3)	52 (67.5)	188 (72.3)
	Fractures	19 (54.3)	33 (42.9)	129 (49.6)
	Aspiration pneumonia	1 (2.9)	5 (6.5)	62 (23.8)
	Stroke	4 (11.4)	22 (28.6)	105 (40.4)
	Ischemic heart disease	4 (11.4)	7 (9.1)	34 (13.1)
	Atrial fibrillation	4 (11.4)	8 (10.4)	24 (9.2)
	Diabetes	8 (22.9)	21 (27.3)	64 (24.6)
	Cancer (active)	0 (0.0)	1 (1.3)	7 (2.7)
	Cancer (history)	7 (20.0)	17 (22.1)	33 (12.7)

Long-term care needs level	1 or 2	13 (37.1)	6 (7.8)	3 (1.2)
	3	12 (34.3)	33 (42.9)	49 (18.8)
	4	6 (17.1)	29 (37.7)	118 (45.4)
	5	4 (11.4)	9 (11.7)	90 (34.6)
Physical disability level (rank)†	J / A	24 (68.6)	34 (44.2)	41 (15.8)
	B1 / B2	7 (20.0)	37 (48.1)	159 (61.2)
	C1 / C2	4 (11.4)	6 (7.8)	60 (23.1)
Cognitive disability level (rank)‡	Independent / I	7 (20.0)	7 (9.1)	13 (5.0)
	IIa / IIb	12 (34.3)	28 (36.4)	53 (20.4)
	IIIa / IIIb	10 (28.6)	25 (32.5)	90 (34.6)
	IV / M	6 (17.1)	17 (22.1)	104 (40.0)

SD, standard deviation

* One missing data

† Based on the "Independence degree of daily living for older adults with disability" are categorized as follows: Rank J: some disabilities, but daily living is mostly independent, capable of going outdoors unassisted; Rank A: indoor living is predominantly independent, but unable to go out without assistance; Rank B: some assistance needed for indoor living, also lies in bed for much of the daytime, although sitting is possible; and Rank C: bedridden all day, requires assistance with excretion/urination, meals, and dressing/undressing (29).

‡ Based on the "Independence degree of daily living for older adults with dementia" are categorized as follows: Independent; Rank I: has some type of dementia, but almost independent in terms of daily living at home and in society; Rank II: some daily life-disturbing symptoms, behaviors and problems in communication seen but can lead daily life independently if watched over by someone; Rank III: daily life-disturbing symptoms, behaviors, and problems in communication that require assistance; Rank IV: daily life-disturbing symptoms, behaviors, and problems in communication that frequently require assistance; and Rank M: marked psychiatric symptoms/related symptoms or serious physical disorders that require expert management (29).

Table S4. Associations Between Frailty Status and Level of Long-term Care Needs: A Sensitivity Analysis Using the Two-category Cut-off Point of ≥8 as Frail

		Frail	Outcome: Frail	
		n / N (%)	Adjusted OR[a]	P value
Level of LTC needs	1 / 2	1 / 22 (4.5)	0.10 (0.01 to 0.79)	0.029
	3	30 / 94 (31.9)	Ref	–
	4	90 / 153 (58.8)	3.07 (1.79 to 5.29)	<0.001
	5	85 / 103 (82.5)	10.66 (5.41 to 20.98)	<0.001

LTC, long-term care; OR, odds ratio

a. The model was adjusted for age (<80, 80–89, and ≥90 years) and sex.
